# Optical coherence tomography analysis of lesion characteristics and thrombus types in non ST-segment elevation myocardial infarction patients

**DOI:** 10.1007/s10554-024-03071-5

**Published:** 2024-06-06

**Authors:** Qing He, Haijia Yu, Jingchao Li, Huihui Song, Luqian Cui, Lingkun Ma, Yue Kang, Zihan Ding, Yingjie Chu, Shujuan Dong

**Affiliations:** Department of Cardiology, Henan Provincial People’s Hospital, Henan University People’s Hospital, Zhengzhou, China

**Keywords:** Non-ST-segment elevation myocardial infarction, Total occlusion, Non-total occlusion, Infarct-related artery, Optical coherence tomography

## Abstract

The precise features of lesions in non-ST-segment elevation myocardial infarction (NSTEMI) patients with total occlusion (TO) of the infarct-related artery (IRA) are still unclear. This study employs optical coherence tomography (OCT) to investigate pathological features in NSTEMI patients with or without IRA TO and explores the relationship between thrombus types and IRA occlusive status. This was a single-center retrospective study. A total of 202 patients diagnosed with NSTEMI were divided into two groups: those with Thrombolysis In Myocardial Infarction (TIMI) flow grade 0 before percutaneous coronary intervention (PCI) (referred to as the TO group, n = 100) and those TIMI flow grade 1–3 (referred to as the Non-TO group, n = 102). Baseline characteristics, coronary angiography findings, and OCT results were collected. Multivariate logistic analysis identified factors influencing TO in NSTEMI. The category of NSTEMI was further subdivided based on the type of electrocardiogram (ECG) into two subgroups: ST segment unoffset myocardial infarction (STUMI) and ST segment depression myocardial infarction (STDMI). This division allows for a more specific classification of NSTEMI cases. The TO group had a younger age, higher male representation, more smokers, lower hypertension and cerebrovascular disease incidence, lower left ventricular ejection fraction (LVEF), and higher creatine kinase myocardial band (CKMB) and creatine kinase (CK) peak levels. In the TO group, LCX served as the main IRA (52.0%), whereas in the Non-TO group, LAD was the predominant IRA (45.1%). Compared to the Non-TO group, OCT findings demonstrated that red thrombus/mixed thrombus was more common in the TO group, along with a lower occurrence of white thrombus (*p* < 0.001). The TO group exhibited a higher prevalence of STUMI (*p* = 0.001), whereas STDMI was more commonly observed in the Non-TO group (*p* = 0.001). NSTEMI presents as STUMI and STDMI distinct entities. Red thrombus/mixed thrombus in IRA often indicates occlusive lesions with STUMI on ECG. White thrombus suggests non-occlusive lesions with STDMI on ECG.

## Introduction

Acute myocardial infarction (AMI) remains the leading cause of morbidity and mortality in patients with cardiovascular disease. ST-segment elevation myocardial infarction (STEMI) and non-ST segment elevation myocardial infarction (NSTEMI) are two types of AMI [1]. The electrocardiogram (ECG) is an essential tool for distinguishing STEMI from NSTEMI. Revascularization is needed when observing ST-segment elevation, the primary ECG indicator requiring immediate attention [2]. However, in clinical practice, a subgroup of patients with total occlusion (TO) of the infarct-related artery (IRA) may exhibit no classical ST-elevation on routine ECG but present with NSTEMI and elevated biomarkers insteadly [3, 4]. According to a study by William C et al. in 2008, occlusive lesions in the IRA were observed in 24% of patients diagnosed with NSTEMI [5]. In a study conducted in 2011, Ino et al. discovered that out of 49 patients with NSTEMI, 29% exhibited red thrombus in the IRA, whereas 39% displayed white thrombus [6]. Vlaar and colleagues (PJ et al.) have also found that a thrombus during the initial angiogram and reduced initial Thrombolysis In Myocardial Infarction (TIMI) flow are associated with retrieving large thrombotic particles, predominantly composed of erythrocytes [7]. In a study conducted by Yu Bo et al. it was found that the different histopathological components of a thrombus may influence the probability of distal embolization unevenly [8–10].

While numerous literature exists on thrombotic types and coronary artery occlusion in NSTEMI patients, most of the studies applied thrombus aspiration and histopathology to explore the components of thrombus. Moreover, research on the specific correlation between occlusive state in the IRA and thrombus types in NSTEMI patients is scarce. This study aimed to examine the variation in thrombotic types between completely occluded and incompletely occluded IRA in patients with NSTEMI. Optical coherence tomography (OCT), the most advanced intravascular imaging technology with 10–20 um resolution, was employed to examine the types of thrombus in the coronary arteries of patients with NSTEMI, both with and without TO of the IRA.

Additionally, we categorized NSTEMI patients into two groups based on the dynamic changes in the ST segment on ECG: ST segment unoffset myocardial infarction (STUMI) and ST segment depression myocardial infarction (STDMI). Our objective was to investigate the association between the occlusive state of the IRA, the various types of thrombus and the ST-segment deviations in NSTEMI patients.

## Materials and methods

### Study population

This study was a single-center retrospective analysis conducted at Henan Provincial People’s Hospital. The patients included in this study suffered NSTEMI and underwent primary PCI and OCT examination between May 2021 and March 2023. NSTEMI is characterized by ischemic chest pain lasting at least 30 min, with elevated biomarkers and dynamic changes. In contrast to STEMI, NSTEMI patients do not manifest persistent ST-segment elevation on an ECG but instead exhibit ST-segment depression and T-wave inversion, or the ECG may be normal [11]. The study excluded patients who met any of the following criteria: (1) Failure to undergo OCT examination or poor imaging quality of OCT; (2) Absence of thrombus detected by OCT; (3) Requirement for surgical coronary artery bypass grafting; (4) Presence of cardiogenic shock; (5) Inability to pass the OCT imaging catheter through the lesions; (6) Presence of restenosis within the stent. This study was approved by the Ethics Committee of Henan Provincial People’s Hospital (approval number: HNSRMYY-2017-47).

### Electrocardiogram

Upon admission, all patients underwent a standard 18-lead ECG and were re-examined three times within 30 min. In NSTEMI, the ST segment of the ECG presents two distinct characteristics: depression and unoffset. To further investigate these manifestations, our research group subcategorized NSTEMI into two subgroups: STUMI and STDMI. STUMI refers to the presence of a stabilized ST segment, with or without accompanying T wave changes, characterized by either a downward shift (ST segment depression) of less than 0.05 mv or an upward shift (ST-segment elevation) of less than 0.1 mv on all leads during the onset. STDMI, on the other hand, is defined as the presence of a new ST-segment exhibiting horizontal or oblique depression of at least 0.05 mv starting from point J on two adjacent leads.

### Coronary angiography

Coronary angiography was conducted by two proficient interventional cardiologists. The examination encompassed the assessment of IRA, lesion location, the number of lesions in vessels, and the TIMI flow grade, all documented in the report. In cases whose reports were inadequate or raised disputes, we reviewed the angiographic disc to verify the data. Furthermore, we clinically ascertained the culprit vessel by considering coronary angiography, changes in ECG and echocardiography integratedly. The TIMI flow criterion was cited to assess the levels of coronary blood flow of IRA in this study [12]. Additionally, we categorized patients with NSTEMI into two subsets: those with a TIMI score of 0 (referred to as the TO group) and those with a TIMI score of 1–3 (referred to as the Non-TO group). To avoid including patients with chronic occlusive disease, we only considered individuals with dynamically increased cardiac biomarkers of AMI.

### Optical coherence tomography

This study performed intracoronary image acquisition using a frequency domain OCT imaging catheter (Optis Mobile OCT Intravascular Imaging System, Abbott Medical). After coronary angiography was completed, an immediate examination of coronary artery OCT should be conducted. In cases exhibited TIMI flow 0–1 in IRA and angiography revealed the presence of thrombus, thrombus aspiration should be performed as a first intervention. Additional small balloon low-pressure pre-dilation was conducted if the TIMI flow remains less than level 2 after thrombus aspiration. Once the TIMI blood flow reaches or exceeds level 2, the OCT imaging catheter was directed along the guide wire towards the distal end of the lesion site responsible for the issue. Two skilled OCT engineers independently assessed the OCT images, blinding to the patient’s angiographic data and clinical presentation. Based on OCT image characteristics, plaque types could be classified into three categories: fibrous, lipid, and calcified plaques [13].

Furthermore, based on the pathological features of plaques, they could be categorized into two types: plaque erosion and plaque rupture [14]. OCT is a precious diagnostic tool which could identify compositions of thrombus in coronary artery of AMI patients. This is achieved by observing specific imaging characteristics associated with the thrombus. The OCT images depict the thrombus as either irregular clusters suspended within the lumen or adhered to the vascular wall, which could be categorized into three distinct types: white, red, and mixed. Red thrombus is characterized by high-backscattering protrusions within the arterial lumen and signal-free shadowing in the OCT image, however white thrombus shows low-backscattering protrusions with signal-rich characteristics [6]. In our study, individuals who presented with only white thrombus on OCT imaging were categorized as having white thrombus, whereas those who showed red thrombus or a combination of red and white thrombus on OCT imaging were classified as having red thrombus/mixed thrombus.

### Clinical data collection

The study collected various clinical baseline data of the included patients, including age, gender, smoking history, hypertension history, diabetes history, cerebrovascular disease history, family history of coronary artery disease (CAD), previous myocardial infarction and angina pectoris, prior history of percutaneous coronary intervention (PCI), biochemical indicators, left ventricular ejection fraction (LVEF), troponin I (TNI) and ischemia time (Ischemia time is calculated as the interval between the patient’s first manifestation of AMI symptoms and the commencement of emergency PCI when balloon dilation achieves a TIMI flow of grade 2 or 3 in the IRA).

### Statistical analysis

The statistical analysis was conducted using SPSS software, version 26.0. Continuous variables were represented as means with their corresponding standard deviation (SD) or medians with their interquartile range (Q1, Q3), depending on their distribution characteristics. A statistical analysis was performed to determine the differences between the two groups. The appropriate tests for this comparison were the Student’s *t*-test and the Mann–Whitney *U* test. They were converted to percentages and compared using either the chi-square test or Fisher’s exact test to analyze categorical variables. A univariate logistic regression was constructed for screening variables that showed significant differences between groups with a *p*-value of less than 0.05. The forward LR stepwise regression method was employed to examine the independent influence of each variable on total occlusion of IRA. Statistical significance in the analysis was determined by a difference with a *p*-value less than 0.05.

## Results

### Baseline characteristics

**Fig. 1:**
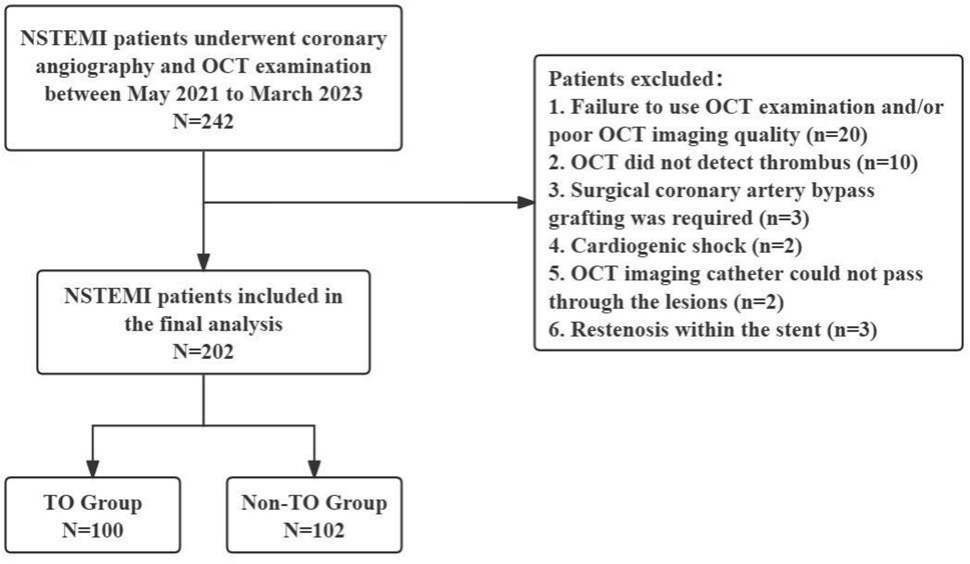
Study flow chart. *NSTEMI* Non ST-segment elevation myocardial infarction, *OCT* Optical coherence tomography, *TO* Total occlusion, *Non-TO* Non total occlusion

After screening based on the inclusion and exclusion criteria (Fig. 1), 202 patients diagnosed with NSTEMI were included in the final analysis. Table 1 presents a comprehensive summary of the baseline characteristics of the patients. Among these cases, 100 (49.5%) were included in the TIMI grade 0 (TO) group, while 102 (50.5%) were included in the TIMI grade 1–3 (Non-TO) group. Compared to patients in the Non-TO group, those in the TO group exhibited a younger age profile and a higher percentage of males. Furthermore, there was a higher prevalence of smoking but a lower incidence of hypertension and cerebrovascular disease among the TO group.

**Table 1:** Comparison of the baseline characteristics between TO group and Non-TO group

Variables	TO group (n = 100)	Non-TO group (n = 102)	*t/z/x*^*2*^	*p* value
Age (years)	56.00 (46.00,64.00)	63.00 (51.00,67.25)	2.893	0.004^*^
Gender			4.583	0.032^*^
Male, n (%)	80 (80.0)	68 (66.7)		
Female, n (%)	20 (20.0)	34 (33.3)		
Coronary artery risk factors				
Smoking, n (%)	62 (62.0)	46 (45.1)	5.798	0.016^*^
Hypertension, n (%)	40 (40.0)	64 (62.7)	10.458	0.001^*^
Diabetes mellitus, n (%)	24 (24.0)	37 (36.3)	3.609	0.057
Dyslipidemia, n (%)	10 (10.0)	4 (3.9)	2.892	0.089
BMI (kg/m^2^)	26.30 (24.20,27.30)	25.60 (24.20,27.00)	1.313	0.189
Family history of CAD, n (%)	36 (36.0)	16 (15.7)	10.900	0.001^*^
Cerebrovascular disease, n (%)	6 (6.0)	16 (15.7)	4.882	0.027^*^
Previous PCI, n (%)	18 (18.0)	10 (9.8)	2.841	0.092
Previous angina pectoris, n (%)	50 (50.0)	42 (41.2)	1.585	0.208
Previous myocardial infarction, n (%)	12 (12.0)	8 (7.8)	0.978	0.323
Killip level on admission			26.757	< 0.001^*^
< ∥, n (%)	57 (57.0)	91 (89.2)		
≥ ∥, n (%)	43 (43.0)	11 (10.8)		
Abnormal left wall motion (%)	67 (67.0)	40 (39.2)	15.647	< 0.001^*^
LVEF (%)	56.50 ± 7.10	61.75 ± 6.20	5.599	< 0.001^*^
Laboratory data				
TG (mmol/L)	1.42 (1.09,2.47)	1.57 (1.18,2.21)	0.231	0.817
CHOL (mmol/L)	4.40 (3.54,5.28)	4.45 (4.06,4.84)	0.366	0.714
HDL-C (mmol/L)	0.99 (0.85,1.20)	1.05 (0.89,1.20)	1.074	0.283
LDL-C (mmol/L)	2.38 (1.99,3.30)	2.76 (2.27,3.07)	0.626	0.531
WBC (× 10^9^/L)	8.63 (6.71,10.60)	8.09 (6.46,9.52)	1.100	0.271
PLT (× 10^9^/L)	201.54 ± 48.09	224.49 ± 61.59	2.948	0.004^*^
HGB (g/L)	146.00 (134.00,155.00)	143.00 (132.00,155.00)	0.674	0.500
CREA (umol/L)	66.50 (57.00,75.00)	62.00 (49.00,70.00)	2.291	0.022^*^
K (mmol/L)	3.89 ± 0.37	4.04 ± 0.42	2.697	0.008^*^
CK peak (IU/L)	1210.00 (594.00,2284.25)	517.00 (180.00,1217.00)	4.999	< 0.001^*^
CKMB peak (U/L)	129.50 (64.25,209.00)	69.00 (28.00,153.00)	4.034	< 0.001^*^
TNI (ng/mL)	0.42 (0.13,1.50)	0.20 (0.06,1.53)	1.787	0.075
Ischemia time (h)	10.00 (6.50,15.88)	9.00 (5.50,15.00)	1.419	0.156

*BMI* Body mass index, *CAD* Coronary artery disease, *PCI* Percutaneous coronary intervention, *LVEF* Left ventricular ejection fraction, *TG* Triglyceride, *CHOL* Total cholesterol, *HDL-C* High-density lipoprotein-cholesterol, *LDL-C* Low-density lipoprotein-cholesterol, *WBC* White blood cell, *PLT* Blood platelet, *HGB* Hemoglobin, *CREA* Creatinine, *K* Kalium, *CK* Creatine kinase, *CKMB* Creatine kinase myocardial band, *TNI* Troponin I,

* Defined as a P-value of less than 0.05, it means that there is a statistically significant difference between the two groups

Additionally, patients in the TO group presented a higher incidence of abnormal left wall motion, elevated creatinine levels, and lower levels of LVEF, potassium, and blood platelet (PLT). Furthermore, the TO group demonstrated higher levels of creatine kinase myocardial band (CKMB) and creatine kinase (CK) levels. It is worth mentioning that no other statistical differences in baseline characteristics were observed.

### Coronary angiography findings

Table 2 presents the coronary angiography findings of the two groups. In the TO group, the left circumflex artery (LCX) was the predominant IRA, accounting for 52.0% of cases. On the other hand, the left anterior descending artery (LAD) was more commonly identified as the culprit vessel in the Non-TO group, constituting 45.1% of cases. In addition, there was a significantly higher occurrence of rentrop grade 1–3 in the TO group compared to the Non-TO group (*p* < 0.001). No other statistically significant distinctions were found between the two groups.

**Table 2:** Comparison of the coronary angiography findings between TO group and Non-TO group

Variables	TO group (n = 100)	Non-TO group (n = 102)	x^2^	*p* value
Culprit coronary artery, n (%)			7.517	0.023^*^
LAD	30 (30.0)	46 (45.1)		
RCA	18 (18.0)	22 (21.6)		
LCX	52 (52.0)	34 (33.3)		
Culprit lesion site, n (%)			3.896	0.143
Proximal segment	56 (56.0)	60 (58.8)		
Mid segment	14 (14.0)	22 (21.6)		
Distal segment	30 (30.0)	20 (19.6)		
CAD lesion, n (%)			1.696	0.428
1-vessel disease	26 (26.0)	26 (25.5)		
2-vessel disease	39 (39.0)	32 (31.4)		
3-vessel disease	35 (35.0)	44 (43.1)		
Bifurcation lesionn, n (%)	44 (44.0)	48 (47.1)	0.190	0.663
Rentrop grade, n (%)			18.278	< 0.001^*^
0	59 (59.0)	86 (84.3)		
1	19 (19.0)	4 (3.9)		
2	14 (14.0)	6 (5.9)		
3	8 (8.0)	6 (5.9)		

*LAD* Left anterior descending, *LCX* Left circumflex, *RCA* Right coronary artery, *CAD* Coronary artery disease, *TIMI* Thrombolysis in myocardial infarction,

* Defined as a P-value of less than 0.05, it means that there is a statistically significant difference between the two groups

### OCT results

Table 3 displays the distribution of thrombus types in the IRA among the 202 NSTEMI patients. Among this cohort, 70 patients had red thrombus/mixed thrombus, while 132 patients had white thrombus in the IRA. Significantly, the patients subject to TO group displayed a higher prevalence of red/mixed thrombus in their IRAs than that in the Non-TO group, whereas the Non-TO group predominantly exhibited white thrombus. Furthermore, the Non-TO group exhibited a higher incidence of microchannel (*p* = 0.019) compared to the TO group. There were no other statistically significant differences identified between the two groups.

**Table 3:** Comparison of the optical coherence tomography results between TO group and Non-TO group

Variables	TO group (n = 100)	Non-TO group (n = 102)	*t/z/x*^*2*^	*p* Value
Plaque morphology, n (%)			0.122	0.727
Fibrous plaque	32 (32.0)	35 (34.3)		
Lipid plaque	68 (68.0)	67 (65.7)		
Thrombus, n (%)			18.578	< 0.001^*^
Red thrombus/mixed thrombus	48 (48.0)	22 (21.6)		
White thrombus	52 (52.0)	80 (78.4)		
Plaque morphology, n (%)			2.862	0.091
Plaque erosion	43 (43.0)	56 (54.9)		
Plaque rupture	57 (57.0)	46 (45.1)		
Macrophages, n (%)	51 (51.0)	58 (56.9)	0.699	0.403
Microchannel, n (%)	2 (2.0)	10 (9.8)	5.504	0.019^*^
Calcification, n (%)	43 (43.0)	34 (33.3)	2.000	0.157
Cholesterol crystal, n (%)	50 (50.0)	57 (55.9)	0.701	0.402
MLA (mm^2^)	1.06 (0.74,1.28)	1.11 (0.74,1.64)	1.575	0.115
Thrombus volume (mm^3^)	1.56 (1.47,1.69)	1.51 (1.26,1.74)	1.709	0.087
Thrombus length (mm)	2.20 (1.80,2.60)	2.20 (1.60,2.40)	1.596	0.11
Thrombus load (%)	25.05 (20.90,27.70)	23.30 (19.65,28.55)	1.652	0.102

*MLA* Minimum lumen area,

* Defined as a P-value of less than 0.05, it means that there is a statistically significant difference between the two groups

### Multivariate logistic analysis

We incorporated variables with a *p*-value < 0.05 from the univariate analysis into the multiple logistic regression model. These variables encompassed age, gender, smoking, hypertension, family history of CAD, cerebrovascular disease, killip level, abnormal left wall motion, LVEF, PLT count, CREA, K, CK peak, CKMB peak, culprit coronary artery, rentrop grade, thrombus and microchannel. The multivariate analysis revealed several factors in the model, including age, Killip level ≥ ∥, smoking, hypertension, family history of CAD, abnormal left wall motion, PLT count, CKMB peak, and white thrombus. Several factors were associated with TO of the IRA. Specifically, Killip level ≥ II, smoking, abnormal left wall movement, a family history of CAD, and elevated peak CKMB levels showed positive associations. Conversely, age, hypertension, PLT count, and the presence of white thrombus were negatively associated with TO of the IRA Table 4.

**Table 4:** Multivariate logistic analysis of factors associated with TO of IRA in NSTEMI

	B	S.E	Wald	Sig	Exp(B)	95% CI for EXP(B)	
						Lower	Upper
Age	− 0.062	0.021	8.834	0.003	0.940	0.903	0.979
Killip level ≥ ∥	1.824	0.524	12.139	0.000	6.196	2.221	17.288
Smoking	0.897	0.408	4.833	0.028	2.453	1.102	5.459
Hypertension	− 1.369	0.407	11.345	0.001	0.254	0.115	0.564
Family history of CAD	1.549	0.487	10.125	0.001	4.706	1.813	12.219
Abnormal left wall motion	1.641	0.469	12.223	0.000	5.158	2.056	12.938
PLT	− 0.010	0.004	4.888	0.027	0.990	0.982	0.999
CKMB peak	0.004	0.002	5.057	0.025	1.004	1.000	1.007
White thrombus	− 1.713	0.449	14.546	0.000	0.180	0.075	0.435
Constant	4.864	1.908	6.502	0.011	129.532		

*CAD* Coronary artery disease, *CKMB* Creatine kinase myocardial band, *PLT* Blood platelet

### Subgroup analysis

**Fig. 2:**
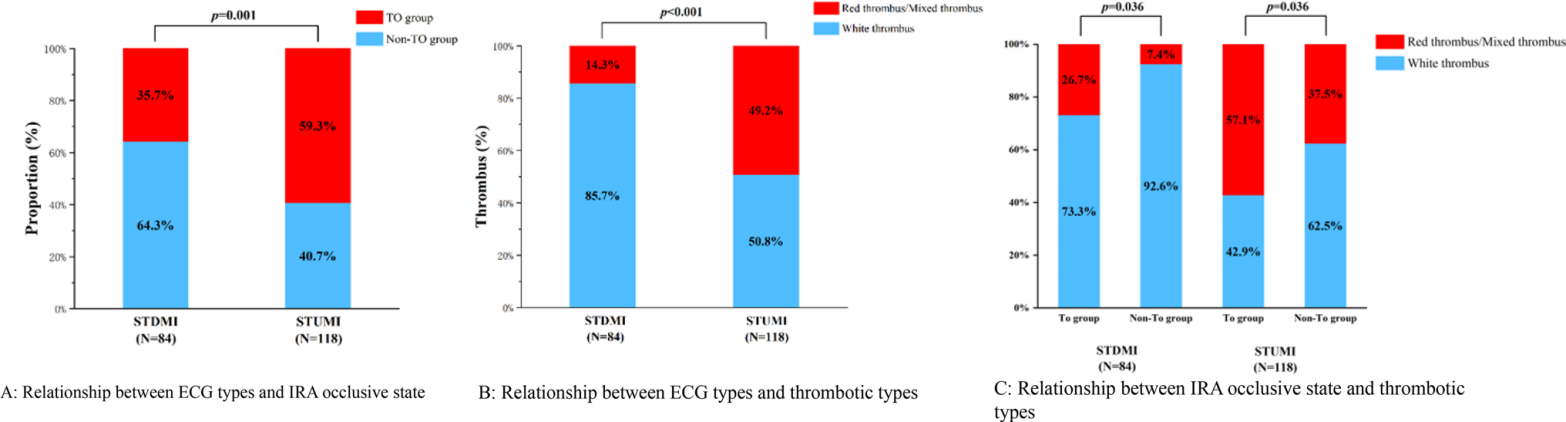
The relationship between thrombotic types and the occlusive state in IRA, based on ST-depression and ST-unoffset on admission ECG

**Fig. 3:**
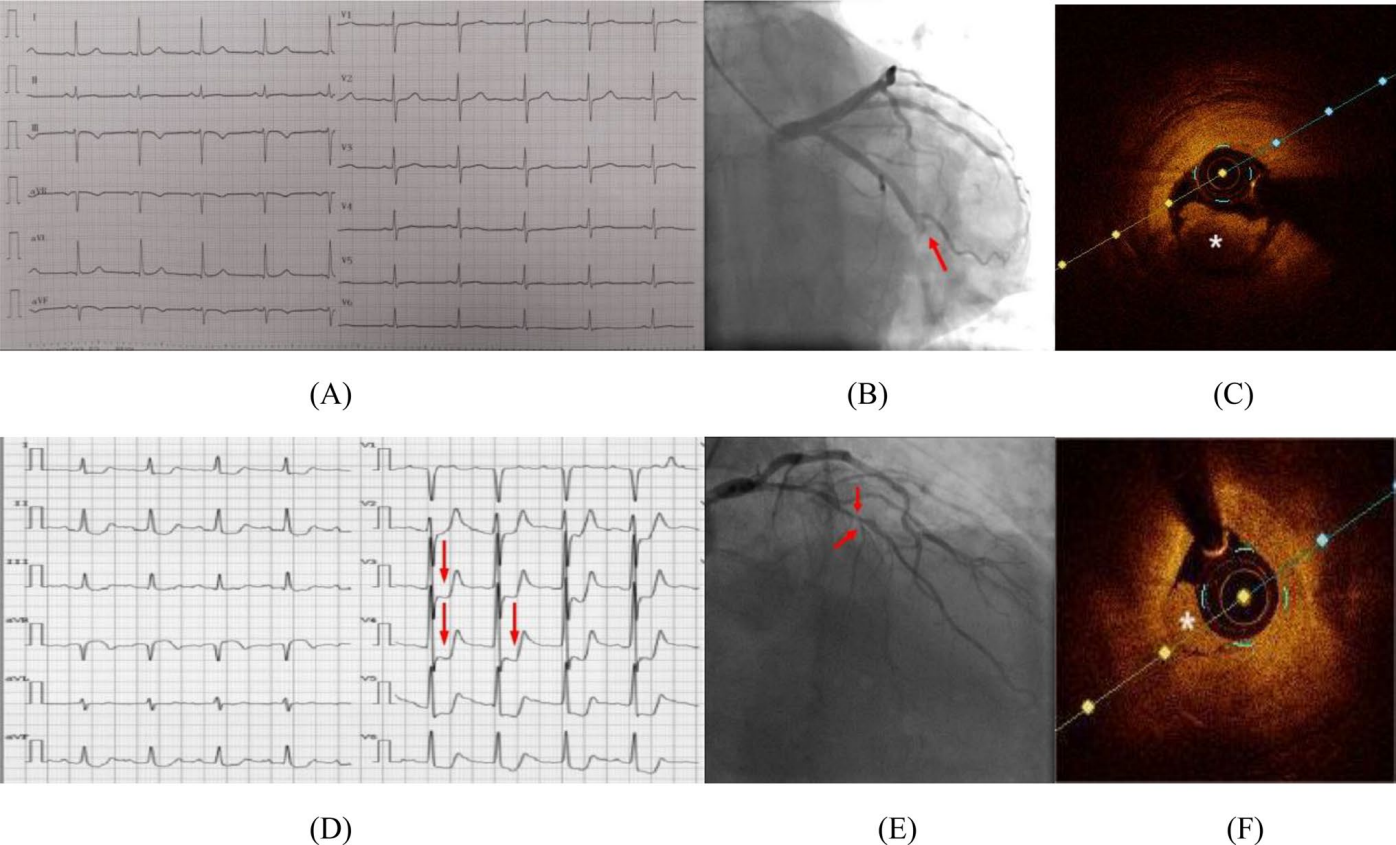
Two representative cases of NSTEMI patients, showcasing their corresponding ECG records, angiography images, and OCT results. Notably, **A**, **B**, and **C** depict the observations made in an elderly male patient who exhibited apparent chest pain symptoms, concurrent elevation in myocardial necrosis markers, and ST-segment unoffset on the initial ECG upon admission. Emergency angiography revealed occlusion of the distal LCX, and OCT result demonstrated the presence of red thrombus (**B** exhibits the occlusive area indicated by the arrow, whereas **C** highlights the site indicated by * as red thrombus). **D**, **E**, and **F** illustrate an older adult displaying noticeable chest pain symptoms. Additionally, he has elevated myocardial necrosis markers and shows ST-segment depression on the initial admission ECG. An urgent angiography showed significant narrowing in the initial and central sections of the LAD, as indicated by the arrow in E. OCT result indicated the presence of white thrombus in the lesion area, in **F**, the site marked by * depicts the location of white thrombus

According to the dynamic alterations observed in the ST segment of their ECGs, we classified all the patients in our study into two subsets: STDMI and STUMI. A total of 202 patients with NSTEMI were included in the study, 84 patients exhibited STDMI on their ECGs, while 118 patients displayed STUMI. The distribution of patients in each group is provided in Fig. 2. According to the information presented in Fig. 2A, it was found that the STUMI ratio was significantly higher in the TO group compared to the Non-TO group, with a significant *p*-value of 0.001. The Non-TO group exhibited predominantly STDMI on their ECGs. Figure 2B presents a comparison of thrombus types between STDMI and STUMI. The results revealed a significantly higher occurrence of red thrombus/mixed thrombus in the STUMI group compared to the STDMI group (*p* < 0.001). Conversely, more white thrombi were observed in the STDMI group compared to the STUMI group (*p* < 0.001). Figure 2C subdivided the ECG types into distinct subgroups, STDMI and STUMI, to explore the relationship between the state of IRA occlusion and different thrombotic types. The integrated analysis exhibited that the ratio of red/mix thrombus was higher than white thrombus in TO group compared with Non-TO group, no matter in STDMI or STUMI subset. This categorization aimed to facilitate a more detailed examination of the association between thrombotic type and the level of occlusion in the IRA. Representative ECG, angiography, and OCT results are illustrated in Fig. 3.

## Discussion

The key findings of this study can be summarized as follows: (1) Approximately 49.5% of NSTEMI patients had complete occlusive lesions in their IRAs. (2) the LCX served as the primary IRA in the TO group, whereas in the non-TO group, the LAD was more frequently identified as the IRA. (3) In this study, over a third of the patients diagnosed with NSTEMI showed the presence of red thrombus/mixed thrombus in their IRAs. Furthermore, this type of thrombus was observed more frequently in NSTEMI patients with TO in the IRA. (4) STDMI and STUMI were identified as two distinct ECG patterns in NSTEMI patients, with the TO group primarily showing STUMI and the non-TO group mainly exhibiting STDMI. (5) NSTEMI patients with red thrombus/mixed thrombus in their IRAs typically displayed STUMI on ECG, whereas those with STDMI predominantly had white thrombus in the IRA.

Our study involved a comparison of the baseline characteristics, angiographic findings, and OCT results between the TO and Non-TO groups. The TO group displayed a younger demographic with a higher frequency of smoking and a lower prevalence of cardiovascular risk factors such as hypertension and diabetes. These findings align with the literature, as reported by Jarosław Karwowski et al. [12]. A study by Wang and colleagues found that 27% of patients diagnosed with non-ST segment elevation acute coronary syndrome (NSTE-ACS) had an occluded infarct artery. Additionally, it was found that these patients exhibited larger infarct sizes and had a higher risk-adjusted 6-month mortality rate [15]. Consistent with these findings, our study revealed a higher proportion of Killip grade ≥ 2, more abnormal left wall motion, and higher peak values of CK and CKMB in the group with TO. Additionally, our literature review revealed that occluded arteries were more prevalent in NSTEMI patients about the LCX. This finding aligned with conventional wisdom, emphasizing that complete occlusion without ST-segment elevation primarily occurs in the LCX. Furthermore, our study observed a higher prevalence of Rentrop grade 1–3 in the TO group. Based on the previous literatures and the experience of our research group, the main reason for acute coronary artery occlusion without ST segment elevation is that the infarcted myocardium has well-developed collateral circulation. This intricate network of blood vessels enables the myocardium affected by ischemia to receive blood supply in a reverse direction, effectively mitigating the impact of the occlusion. Furthermore, the absence of ST-segment elevation in patients with occlusion of the distal end of the LCX can be attributed to the fact that the location of the lower posterior wall of the heart is a blind spot detected by a 12-lead or even 18-lead ECG [16–18].

The current guidelines for managing acute coronary syndrome (ACS) emphasize the emergency revascularization of IRA for patients who present with STEMI [19]. However, in cases with no typical ECG indications, there is a potential risk of underestimating the severity of certain NSTEMI patients with total occlusion of the infarct-related artery (TO of IRA). Consequently, the delay or even exclusion of revascularization procedures may occur. A study conducted by Abdul R et al. (2017) demonstrated that patients diagnosed with NSTEMI who experienced total occlusion of the IRA, as observed during coronary angiography, were associated with a significantly higher mortality risk and major adverse cardiac events. Early vascular reconstruction could potentially enhance the prognosis [2]. In 2016, Jaroslaw Karwowski et al. conducted a study on a cohort of 2767 NSTEMI patients in Poland who had been registered for ACS. The researchers discovered that 26.3% of the patients had TIMI flow grade 0, while the remaining 73.7% had TIMI flow grade 1–3 [12]. In a more recent study conducted in 2021, Irmina Morawska et al. included 399 NSTEMI patients in their research, according to the findings, 138 were classified as having preoperative TIMI flow grade 0 (TO group), while 261 patients fell into the preoperative TIMI flow grade 1–3 category (Non-TO group) [20]. There is a rising trend of individuals with NSTEMI who experience TO of the culprit artery. Our study revealed that almost half (49.5%) of NSTEMI patients had TO of the culprit artery, a considerably higher proportion than previous literature. Hence, it is crucial to recognize and prioritize the NSTEMI population presenting with TO of the culprit artery, and further research is necessary to enhance the identification of this particular group.

According to traditional theory, STEMI is commonly caused by a single vessel occlusive lesion, characterized by red blood cells and fibrinogen as the primary components of the thrombus. The treatment approach for STEMI focuses on wholly and promptly opening the IRA while simultaneously implementing intensified antithrombotic and anti-ischemic measures. Thrombolysis or emergency intervention may also be viable treatment options. In contrast, NSTEMI typically presents as a non-occlusive lesion involving multiple blood vessels, with platelet representing the major thrombus component. The recommended treatment approach for NSTEMI involves enhancing antithrombotic and anti-ischemic measures, and if necessary, emergency intervention can be considered. However, thrombolysis is contraindicated for NSTEMI. In 2012, Quadros et al. performed a pathological examination on thrombi obtained from the affected blood vessels of 113 patients diagnosed with STEMI. Their study revealed that approximately one-third (31%) of the thrombi observed in STEMI patients appeared as white thrombi [21]. In 2011, Yasushi et al. employed OCT technology to investigate thrombus formation in the IRA of 89 patients diagnosed with ACS. The study population comprised 40 cases of STEMI and 49 cases of NSTE-ACS. Their study revealed that in STEMI patients, 78% had red thrombus in the IRA, whereas 22% had white thrombus in the IRA. Additionally, among NSTE-ACS patients, 39% had white thrombus, and 27% had red thrombus in the IRA [6]. These findings suggest that red thrombus is more prevalent in STEMI cases, while white thrombus is more common in NSTE-ACS. Consistent with prior literature, the NSTEMI population in which the IRA primarily consists of white thrombus was examined. Within our study, encompassing 202 NSTEMI patients, we uncovered 132 white thrombus and 70 cases of red thrombus. Among the 70 cases of red thrombus, occlusive lesions were present in 48 cases (68.6%), and non-occlusive lesions were present in 22 cases (31.4%) in the IRA. Our research reveals that red thrombus predominantly occurs in the group with TO of the IRA, whereas white thrombus exists primarily in the group without total occlusion (Non-TO) of the IRA. Based on these findings, we hypothesize that blood flow is a crucial factor influencing the type of thrombus and that the type of thrombus is correlated with the TIMI flow of the IRA. The association between ST-segment elevation and thrombotic composition is correlative rather than causal. Hence, the understanding that “ST segment elevation indicates red thrombus” and “ST segment non-elevation indicates white thrombus” lacks rigor. Consequently, adopting a “one-size-fits-all” treatment approach for AMI patients is not justifiable solely based on ECG manifestations. Based on the study’s findings, a hypothesis suggests a significant correlation between the type of thrombus and the occurrence of coronary artery occlusion. The study reveals that patients with primarily red thrombus in the IRA tend to have occlusive lesions, while those with primarily white thrombus in the IRA tend to have non-occlusive lesions. We innovatively used OCT technology to observe coronary thrombus in NSTEMI patients, Luis Augusto Palma Dallan et al. found that OCT-proven images completely changed the treatment of patients, mainly manifested by further improvement of treatment plans, including adequate pre-dilation and ideal stent size with good treatment outcomes [22]. If the IRA shown by our OCT are red thrombus/mixed thrombus, whether the one-size-fits-all treatment strategy can be interrupted to perform thrombolytic therapy for these NSTEMI patients is a question worth exploring.

The ST segment of the ECG in patients with NSTEMI comprises two distinct characteristics: depression and unoffset. Are there any similarities or differences between these characteristics, similar to the distinctions between NSTEMI and STEMI? In a 2021 study by Shujuan Dong et al., a group of individuals diagnosed with occlusion of the IRA were analyzed and divided into three distinct categories according to their ST segment characteristics: ST-segment elevation, normal ST segment, and ST segment depression. The study findings suggested that patients with myocardial infarction characterized by ST-segment elevation or a normal ST segment frequently exhibited more occlusive lesions [23]. Consistent with our study, we observed that NSTEMI patients with STUMI (normal ST segment) showed a higher occurrence of occlusive lesions than those with STDMI (ST segment depression). Furthermore, our investigation revealed discrepancies in the thrombotic types between STUMI and STDMI as observed by OCT. Patients with red thrombus/mixed thrombus in their IRA typically displayed STUMI on ECG, whereas patients with STDMI predominantly displayed white thrombus in the IRA. In the case of NSTEMI, some patients may benefit from thrombolysis treatment. It is arbitrary to assess the suitability of ST segment elevation for thrombolysis in a biased manner.

The findings of this study subdivided NSTEMI into STUMI and STDMI, deepening the cognitive theory of NSTEMI. Additionally, our study demonstrated the presence of a substantial number of red thrombi in the IRA of NSTEMI patients, with a majority observed in TO of the IRA. These findings provide a valuable research foundation for further refinement of NSTEMI treatment strategies. (clinical implication).

### Limitations

This study is limited in a few ways. Firstly, it is a retrospective analysis conducted at a single center, which might restrict the generalizability of the findings. Additionally, the sample size in this study is relatively small, which could reduce the statistical power and limit the generalizability of the results. To address these limitations, future studies should consider conducting prospective multi-center trials with larger sample sizes to enhance the validity and generalizability of the findings. Secondly, a few NSTEMI patients were excluded during the study period, including those with restenosis within the stent. The presence of the steel beam in the stent may have impacted the evaluation of plaque and thrombus characteristics using OCT. Additionally, some patients did not find thrombus through OCT, which might have introduced selection bias that could have influenced the results. Thirdly, in NSTEMI patients with TIMI grade less than 2, it is imperative to conduct a small balloon pre-dilation before OCT to ensure adequate blood perfusion. This pre-dilation process, however, carries the risk of potential mechanical damage, which in turn could lead to alterations in the morphological features of potential plaques in these patients. Lastly, it is essential to validate the findings of this study through large-scale, multicenter clinical investigations.

## Conclusion

In NSTEMI, STUMI and STDMI are two distinct objective entities. Red thrombus/mixed thrombus in IRA often indicates occlusive lesions with STUMI on ECG. On the other hand, non-occlusive lesions, mainly associated with white thrombus, are prevalent in IRA of most patients, with their ECGs predominantly displaying STDMI.

## Data Availability

Shujuan Dong could be contacted if someone wants to request the data from this study.
